# Urbanization change in a mega-event preparation context: A multidimensional assessment of Zhangjiakou, a medium-sized co-host city of the Beijing 2022 Winter Olympics

**DOI:** 10.1371/journal.pone.0339708

**Published:** 2026-05-06

**Authors:** Lemeng Liu, Wei Zhang, Jinghua Long, Jintian Yang, Wentong Jia

**Affiliations:** 1 School of Public Administration, Hebei University of Economics and Business, Shijiazhuang, China; 2 Design School, Xi’ an Jiaotong-Liverpool University, Suzhou, China; 3 Hebei Collaborative Innovation Center for Urban-rural Integrated Development, Shijiazhuang, China; 4 College of Physical Education, Hebei Normal University, Shijiazhuang, China; Tsinghua University, CHINA

## Abstract

Urbanization is a key pathway for regional transformation and an important lens for assessing the legacy of mega sporting events. Focusing on Zhangjiakou City, co-host city of the Beijing 2022 Winter Olympics, this study constructs a multidimensional urbanization index covering population, economy, built-environment, and ecology using the CRITIC weighting method, and applies spatial autocorrelation analysis and GeoDetector to examine urbanization dynamics from 2017 to 2022. The main findings are as follows: (1) Zhangjiakou’s total urbanization level increased by 20.68%; population, economic, and spatial urbanization improved, whereas ecological urbanization followed an inverted-U trajectory and declined by the end of the study period. (2) High-level urbanization areas expanded from the central urban core toward the northern region, and the share of districts/counties at medium level or above increased markedly. (3) Economic urbanization showed significant positive spatial autocorrelation, with high-high clusters concentrated in the central urban area and later extending to Zhuolu County. (4) The economic dimension consistently showed the strongest explanatory power, while urban economic density, per capita urban road area, population density distribution, and total urban population were the most stable explanatory factors. These findings show multidimensional and spatially uneven urbanization during the Olympic preparation period in a medium-sized co-host city.

## 1. Introduction

Mega sporting events are widely regarded as important catalysts of urban transformation in host cities [1]. Their impacts are often reflected in enhanced city visibility, economic restructuring, infrastructure investment, tourism development, and cultural exchange [2–6]. Urbanization provides a useful lens through which to examine these transformations because it is a multidimensional process involving demographic concentration, economic growth, spatial expansion, infrastructure upgrading, and environmental change [7–11]. From this perspective, mega-event preparation may reshape not only the scale of urban development, but also its composition, geography, and sustainability. At the same time, the urban consequences of mega sporting events are not uniformly positive. Existing studies have shown that while such events may stimulate urban growth and modernization, they may also generate displacement, socio-spatial inequality, debt burdens, and environmental pressure [12–14]. The relationship between mega-event preparation and urbanization therefore remains complex, multidimensional, and highly context-dependent.

A substantial body of research has examined the ways in which mega sporting events may contribute to urbanization. From a demographic perspective, large sports venues and event preparation can influence population agglomeration, migration flows, and labour redistribution [15–16]. From an economic perspective, mega-events may promote industrial upgrading, attract investment, and generate new development opportunities in tourism, services, and related sectors [17–19]. In terms of urban construction and spatial development, many host cities have benefited from transport upgrading, real-estate development, and expanded urban infrastructure during event preparation [20–22]. Some studies further suggest that mega-events may create opportunities for environmental governance, low-carbon transition, and urban greening, especially when sustainability principles are incorporated into planning and investment strategies [23–26]. Taken together, these studies indicate that mega-events may act as important stimuli for urban change.

However, other studies have highlighted the uneven and sometimes negative consequences of mega-events. In some cases, event-led restructuring has been associated with labour loss, industrial decline, or unstable post-event development [27]. Large-scale infrastructure construction and venue development may intensify socio-economic inequality, induce population displacement, and contribute to housing affordability problems [28–29]. Financially, several mega-events have imposed substantial fiscal pressure on host cities, limiting their long-term development capacity [30]. Environmentally, even where sustainability programmes are introduced during preparation, their effects may be temporary or undermined by broader political and economic constraints [31]. Taken together, these contrasting findings suggest that the key issue is not simply whether mega-events affect urbanization, but how the gains and costs of event preparation are distributed across dimensions and across space.

Although previous studies have generated important insights, three limitations remain. First, much of the literature focuses on core metropolitan hosts or national capitals, while relatively limited attention has been paid to medium-sized co-host cities located in metropolitan peripheries. Yet such cities are particularly important because they are often expected to absorb event-related investment, infrastructure, and tourism growth without possessing the same economic base, administrative capacity, or spatial advantages as major metropolitan centres. Second, many existing studies focus on one or two outcomes, such as GDP growth, tourism development, land expansion, or housing markets, rather than examining urbanization as a multidimensional process involving population, economy, built environment, and ecology. This makes it difficult to assess whether event preparation produces balanced urban transformation or uneven gains across dimensions. Third, relatively little evidence is available at the district- and county-level to show how the benefits and costs of mega-event preparation are distributed within host cities. Intra-urban heterogeneity is especially important in large and territorially diverse cities, where some areas may benefit strongly from new infrastructure and investment while others remain marginal to event-led development.

Methodologically, previous research has relied mainly on statistical analysis, interviews, and case studies to investigate the urban effects of mega sporting events [32–34]. These approaches have made important contributions, but they may be less effective in capturing fine-scale spatial heterogeneity, especially in areas where official statistics are limited or uneven in quality. With the rapid development of satellite observation technology, remote sensing data have become increasingly valuable for urban studies because they provide continuous, spatially explicit, and timely information that is difficult to obtain from conventional statistical records alone [35–36]. Remote sensing therefore plays a crucial role in compensating for the limitations of conventional statistical data in small-area studies.

Taken together, existing studies leave an important analytical question unresolved: how does mega-event preparation reshape urbanization across multiple dimensions and across space within a medium-sized co-host city, rather than in a core metropolitan host?

Zhangjiakou provides a particularly informative case for addressing these gaps. As a co-host city of the Beijing 2022 Winter Olympics, it is not a global metropolis in its own right, but a medium-sized city located on the Beijing metropolitan periphery and within the Beijing-Tianjin-Hebei coordinated development region. Its proximity to Beijing, improving transport connectivity, and growing role in regional development provide important urbanization opportunities, including access to wider markets, infrastructure upgrading, and tourism spillovers. At the same time, Zhangjiakou faces substantial development constraints, including a relatively weak economic base, uneven county-level development, limited population agglomeration capacity, and considerable ecological sensitivity. These characteristics make it especially suitable for examining how mega-event preparation interacts with pre-existing urbanization potential and uneven regional development conditions, rather than assuming a simple direct Olympic effect. Moreover, compared with core metropolitan hosts, Zhangjiakou makes it possible to explore whether event-related urbanization in a medium-sized co-host city is broad-based, spatially selective, or accompanied by ecological trade-offs.

This study examines multidimensional urbanization dynamics in Zhangjiakou during the preparation period for the Beijing 2022 Winter Olympics. Specifically, it addresses three questions: (1) How did multidimensional urbanization in Zhangjiakou change from 2017 to 2022 during the Winter Olympics preparation period? (2) How were these changes distributed spatially across districts and counties? (3) Which dimensions and indicators were most strongly associated with the spatial heterogeneity of urbanization? To answer these questions, the study integrates remote sensing data and socio-economic statistics to construct a multidimensional urbanization evaluation framework covering population, economy, built environment, and ecology. Within this framework, the CRITIC method is used to derive data-driven weights for heterogeneous indicators, spatial autocorrelation analysis is employed to identify clustering patterns, and GeoDetector is applied to assess explanatory associations. Rather than estimating the isolated causal effect of hosting the Winter Olympics, the paper focuses on urbanization dynamics during the Olympic preparation period within a specific regional development context.

This paper makes three main contributions. First, it extends the mega-event urbanization literature from core metropolitan hosts to a medium-sized co-host city on the Beijing metropolitan periphery, thereby highlighting how event-related urbanization is mediated by uneven regional development conditions. Second, it conceptualizes urbanization as a multidimensional process spanning population, economy, built environment, and ecology, and shows that gains in some dimensions may coexist with ecological decline and spatially uneven outcomes. Third, by integrating multi-source remote sensing and statistical data at the district- and county-level, it provides a spatially explicit account of how infrastructure provision, economic agglomeration, and local development conditions shaped urbanization heterogeneity during the Olympic preparation period. More broadly, the study contributes to debates on the uneven gains and environmental trade-offs of mega-event-led urban transformation.

## 2. Materials and methods

### 2.1 Study Area

Zhangjiakou City is located in northwestern Hebei Province, China, covering approximately 36,800 km^2^ (113°50′-116°30′ E, 39°30′-42°10′ N). It forms an important gateway linking Beijing, northern Hebei, and Inner Mongolia. As a medium-sized city on the Beijing metropolitan periphery and within the Beijing-Tianjin-Hebei coordinated development region, Zhangjiakou occupies a strategic position in regional transport, tourism, and resource flows. Its proximity to Beijing provides access to a large surrounding market and creates opportunities to benefit from capital spillovers in terms of infrastructure investment, visitor demand, and industrial linkages. In particular, the upgrading of transport connections during the Winter Olympics preparation period, including high-speed rail and road infrastructure, further improved its regional accessibility.

**Fig 1:**
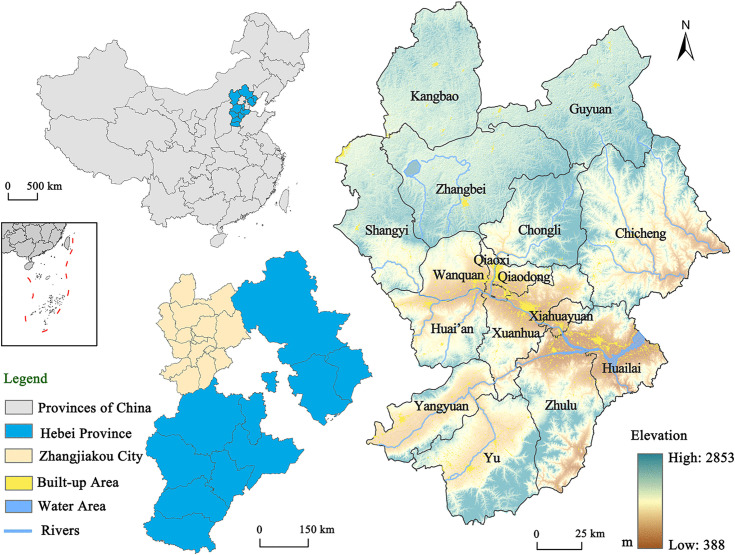
Location map of Zhangjiakou City. This map is created using ArcGIS 10.8 (https://desktop.arcgis.com/en/arcmap/latest/get-started/main/get-started-with-arcmap.htm). The administrative boundary vector of the study area are sourced from the Environmental Science and Data Center, CAS (https://www.resdc.cn/). The built-up area and the Digital Elevation Model (DEM) are from the China Land Cover Dataset (CLCD) (https://code.earthengine.google.com/77ed04f1ad0f5dea08601eef9e01199f) and the MODIS terrain database (https://code.earthengine.google.com/cbee1df6686a326ee1459189782879b9) of Google Earth Engine, respectively.

At the same time, Zhangjiakou has long faced structural development constraints. Compared with core metropolitan areas, its economic base is relatively weak, its industrial structure has historically depended on traditional sectors, and its counties display marked internal disparities in development conditions. Population agglomeration capacity is also comparatively limited, and some areas have experienced development pressure associated with out-migration and uneven urban growth. In recent years, the city has sought to cultivate new growth drivers, including winter tourism, renewable energy, and digital industries. However, its mountainous terrain, ecological fragility, and environmental protection requirements mean that urban expansion and infrastructure construction are subject to significant spatial and ecological constraints. This combination of locational advantages, policy opportunities, developmental limitations, and environmental sensitivity makes Zhangjiakou an appropriate case for analysing multidimensional urbanization dynamics during the preparation period for the Beijing 2022 Winter Olympics (Fig 1).

### 2.2 Data Source

This study integrates remote-sensing products and statistical yearbooks to quantify multi-dimensional urbanization in Zhangjiakou from 2017 to 2022 (Table 1).

**Table 1:** Data source.

Data	Resolution	Source
Administrative boundary vector	–	The Environmental Science and Data Center, Chinese Academy of Sciences (https://www.resdc.cn/)
Statistical data		Zhangjiakou Yearbook (2017–2022) and Hebei Economic Yearbook (2017–2022) (https://data.cnki.net/)
NDVI	10m	Google Earth Engine (https://code.earthengine.google.com/711771f41a350738806c97f58e211258)
API	1km	Google Earth Engine (https://code.earthengine.google.com/d55e32996dd8f4eddaed9284eac3fc5e?noload=1)
Nightlight data	1km	NPP/VIIRS satellite dataset of the Institute of Geographic Sciences and Natural Resources Research, Chinese Academy of Sciences (https://www.resdc.cn/DOI/DOI.aspx?DOIID=106)
Population	1km	Worldpop database of the University of Southampton, United Kingdom (https://hub.worldpop.org/)
Building footprint vector data	2.5m/0.5m	CBRA multi-annual rooftop-area dataset (https://doi.org/10.5281/zenodo.7500612) and East Asia building footprint vector dataset (https://doi.org/10.5281/zenodo.8174931)
Building Height	10m	CNBH-10m dataset (https://zenodo.org/records/7827315)

Remote-sensing data. NDVI and Annual air-pollution indicators were respectively derived from Sentinel-2 and Sentinel-5P imagery using Google Earth Engine. The Sentinel-5P API weighting scheme follows Wang et al. (2022), with the weighting rationale derived from Campos et al. (2021) [37,38]. Nightlight data were obtained from the NPP/VIIRS dataset provided by the Institute of Geographic Sciences and Natural Resources Research, Chinese Academy of Sciences, and were pre-processed following [39]. Population density data were obtained from the WorldPop database (University of Southampton, UK). Building-height data were obtained from the CNBH-10m dataset, which estimates building height (m) by integrating multi-source remote-sensing imagery and digital surface model data via machine-learning modeling [40]. In this study, CNBH-10m was used as a static height baseline, and year-specific building/built-up boundaries were used to clip the height layer to construct height-informed built-space proxies for annual comparison. Building footprint/boundary data for 2017–2021 were derived from the CBRA multi-annual rooftop-area dataset (2.5 m; 2016–2021) [41]. For 2022, building footprints were obtained from the 0.5m East Asia building footprint vector dataset released by Shi et al. (2024) [42], which was used as the most up-to-date footprint layer available for the study area.

Statistical data. Socioeconomic and infrastructure variables (e.g., per capita park green area, per capita urban road area, urban economic density, GDP per capita, total urban population, non-agricultural employment share, Engel coefficient, disposable income of urban residents, and the ratio of environmental protection investment to total investment) were collected from the Zhangjiakou Yearbook (2017–2022). Additional variables (e.g., urbanization rate, tertiary-industry share, and energy consumption per 10,000 RMB of GDP) were collected from the Hebei Economic Yearbook (2017–2022).

### 2.3 Constructing the urbanization evaluation index system based on multi-source data

Urbanization is conceptualized here as a multidimensional process involving population concentration, economic agglomeration, built-environment expansion, and ecological change. Accordingly, four dimensions: population, economy, built-environment, and ecology, were selected because they represent major structural components of urbanization frequently discussed in the literature. At the same time, these dimensions can be operationalized relatively consistently at the district- and county-level using available remote sensing and socio-economic data, which is essential for examining small-area spatiotemporal dynamics from 2017 to 2022. Nevertheless, this framework represents only a partial interpretation of urbanization and does not directly capture other important dimensions such as governance capacity, public service provision, social inequality, housing affordability, or subjective well-being.

**Fig 2:**
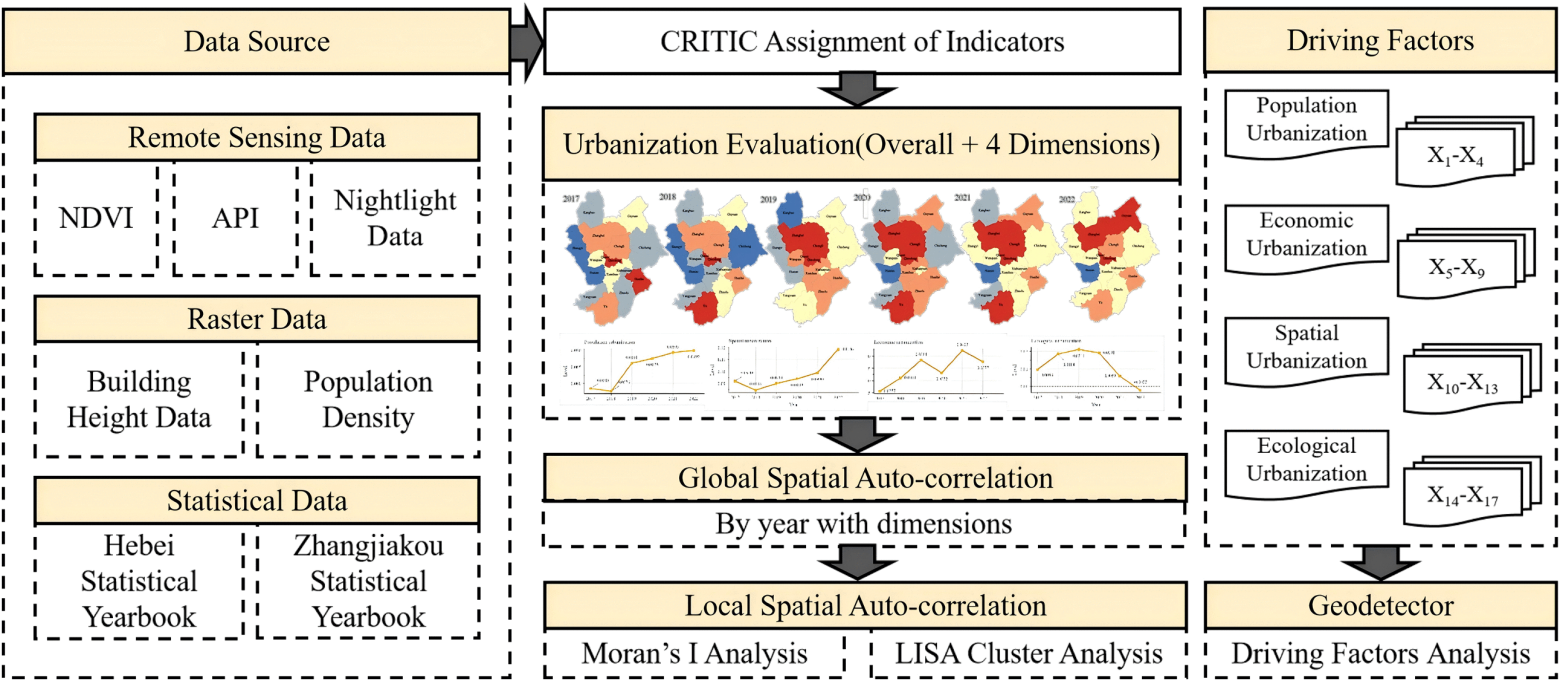
Data processing framework. The administrative boundary vector data used in this figure are from the Resource and Environment Science and Data Center, Chinese Academy of Sciences.

To operationalize these dimensions, this study constructs a multi-source urbanization evaluation index system and examines the urbanization dynamics of Zhangjiakou during 2017–2022 in the context of the Winter Olympics preparation period. For the population dimension, WorldPop gridded population data are used to provide spatially explicit population distribution with greater temporal continuity than conventional statistics. For the economic dimension, nighttime light imagery is adopted as an objective proxy for the intensity of regional economic activity. For the built-environment dimension, built-up area captures horizontal expansion but cannot reflect vertical development; therefore, a height-informed built-space proxy is introduced by integrating building-height information with annual building/built-up boundaries to approximate three-dimensional development intensity. For the ecological dimension, Sentinel-5P atmospheric composition products are used to characterize the spatial patterns of key air pollutants as a consistent remote-sensing-based indicator of environmental conditions. The overall data-processing workflow is shown in Fig 2.

After zonal statistics were computed for the remote-sensing data, all indicators were normalized, weighted using the CRITIC method (Table 2) [43], and aggregated by weighted summation to obtain the final evaluation results [44]. The CRITIC method was selected to determine indicator weights in a data-driven manner. Compared with expert-based approaches such as AHP, which rely on pairwise judgments to derive priorities, CRITIC reduces subjectivity when the purpose is to summarize observed multidimensional variation rather than to encode normative preferences [45,46]. It was also preferred over simpler objective weighting schemes because the present framework combines heterogeneous remote-sensing and socio-economic indicators that differ not only in variance but also in potential redundancy [47,48]. In particular, equal weighting does not account for differences in informational contribution across indicators, while entropy-based weighting methods primarily emphasize contrast intensity but do not explicitly account for inter-indicator conflict or redundancy [49,50]. By contrast, CRITIC incorporates both indicator variability and inter-indicator correlation, assigning greater weight to indicators that provide more distinctive information. This makes it particularly suitable for constructing a multidimensional urbanization index in Zhangjiakou, where several indicators may partly capture related aspects of population, economic, built-environment, and ecological change. In this study, CRITIC is used specifically to construct the composite urbanization index, while spatial autocorrelation analysis and GeoDetector are subsequently employed to interpret spatial clustering and explanatory associations.

**Table 2:** Urbanization evaluation system.

Target Layer	Dimension Layer	Indicator Layer	Indicator Code	Unit	Weight	Attribute
Urbanization Level	Population Urbanization	Population Urbanization Rate	X_1_	%	0.0540	+
		Population Density Distribution	X_2_	–	0.0464	+
		Total Urban Population	X_3_	Person	0.0502	+
		Percentage of Non-Agricultural Employment	X_4_	–	0.0695	+
	Economic Urbanization	Per Capita GDP	X_5_	RMB	0.0454	+
		Percentage of Output Value of the Tertiary Industry	X_6_	%	0.0498	+
		Engel Coefficient	X_7_	–	0.0618	–
		Disposable Income of Urban Residents	X_8_	RMB	0.0471	+
		Night Light Data	X_9_	–	0.0527	+
	Spatial Urbanization	Per Capita Estimated Floor-space Proxy	X_10_	m^2^	0.0616	+
		Per Capita Green Park Area	X_11_	m^2^	0.0531	+
		Per Capita Urban Road Area	X_12_	m^2^	0.0831	+
		Urban Economic Density	X_13_	10,000 RMB/km^2^	0.0535	+
	Ecological Urbanization	NDVI	X_14_	–	0.0670	+
		Annual Average API	X_15_	–	0.0846	–
		Energy Consumption per 10,000 RMB of GDP	X_16_	tce/10,000 RMB	0.0573	–
		Ratio of Government Environmental Protection Investment to Total Investment	X_17_	%	0.0628	+

The normalization formulas are as follows:

Positive indicators:


$$x_{ij}=\frac{P_{ij}-min(P_{ij})}{max(P_{ij})-min(P_{ij})}$$ (1)

Negative indicators:


$$x_{ij}=\frac{max(P_{ij})-P_{ij}}{max(P_{ij})-min(P_{ij})}$$ (2)

In Equations (1) and (2), $P_{ij}$ is the original index value, *x*_*ij*_ is the index value after standardization, and *P* is the threshold value.

A strength comparison between indicators is performed as shown in Equation (3):


$$\left\{ \begin{array}{c} \overset{―}{x_{j}}=\frac{\text{1}}{n}\sum_{i=\text{1}}^{n}x_{ij}\;\\ s_{j}=\sqrt{\frac{\sum_{i=\text{1}}^{n}{\left(x_{ij}-{\overset{―}{x}}_{j}\right)}^{\text{2}}}{n-\text{1}}} \end{array}\right.\;$$ (3)

Equation (3) aims to determine the mean of indicator *j*, *S*_*j*_ represents the standard deviation of indicator *j*.

An indicator conflict analysis is performed as shown in Equation (4):


$$R_{j}=\sum_{i=\text{1}}^{p}\left(\text{1}-r_{ij}\right)$$ (4)

*R*_*j*_ is the degree of conflict between indicator j and the other indicators, 0 < *r*_*ij*_ < 1.

Information volume calculation is performed as shown in Equation (5):


$$C_{j}=S_{j}\sum_{i=\text{1}}^{p}(\text{1}-r_{ij})=S_{j}\times R_{j}$$ (5)

Weight calculation is performed as shown in Equation (6):


$$W_{j}=\frac{C_{j}}{\sum_{j=\text{1}}^{P}C_{j}}$$ (6)

*W*_*j*_ represents the weight of indicator *j*.

The comprehensive index, i.e., the urbanization evaluation score, is calculated as shown in Equation (7):


$$Q=\sum_{u=1}^{n}h_{u}\times r_{u}$$ (7)

Here, *Q* is the comprehensive evaluation score of the evaluation system, *u* is the evaluation index, *h*_*u*_ is the evaluation index value, and *r*_*u*_ is the corresponding evaluation index weight. In this study, the urbanization evaluation result is obtained by multiplying the result obtained by the dimensionless index data and weight.

### 2.4 Height-informed built-space proxy

To characterize the vertical built-environment consistently over 2017–2022, we derived height-informed built-space indicators by combining a fixed high-resolution building-height layer with year-specific built-up extent masks.

Let *H*_*2020*_(*x*) denote building height (m) at pixel *x* from the CNBH-10m (2020) dataset, and let *B*_*t*_ denote the binary mask of the building (or built-up) extent for year t (t ∈ {2017,...,2022}), where *B*_*t*_(*x*)= 1 indicates pixels inside the year-t boundary and *B*_*t*_(*x*)= 0 otherwise. Let $A_{x}$ be the pixel area (m^2^). For an analysis unit *u*, indicators are computed over pixels *x* ∈ *u*.

Storey-height assumption. When interpreting height in terms of an approximate number of storeys, we use 2.8 m per storey, consistent with the Design Code for Residential Buildings (GB 50096−2011), which specifies that residential storey height is typically 2.80 m. Accordingly, an approximate storey proxy can be expressed as $S_{2020}(x)=H_{2020}(x)/2.8$.

Mean height within the year-specific boundary (height structure proxy) (Equation (8)):


$${\bar{H}}_{t}=𝐦𝐚𝐱\left(H_{2020}\left(x\right)|B_{t}\left(x\right)=1\right)$$ (8)

Height-informed built-space supply proxy (areal “volume” proxy) (Equation (9)):


$${𝐕}_{𝐭}=\sum_{𝐱\in {𝐁}_{𝐭}}^{-}{𝐇}_{2020}\left(𝐱\right)\cdot {𝐀}_{𝐱}$$ (9)

where *A*_*x*_ is the pixel area (m^2^). *V*_*t*_ can be interpreted as an areal proxy for built space supply that accounts for vertical development (i.e., a height-weighted built up area).

Per-capita proxy (Equation (10)):


$$\overset{𝐩𝐜}{\underset{𝐭}{{𝐕}_{𝐭}}}=\frac{{𝐕}_{𝐭}}{{𝐏}_{𝐭}}$$ (10)

where Pt is the population of the unit in year *t*.

Importantly, these indicators do not represent officially reported floor area or per-capita housing area. Instead, they serve as relative, height-informed proxies of built-environment structure and its inter-annual variation, which is driven primarily by changes in the mapped built-up extent *B*_*t*_, under the assumption that vertical morphology is relatively stable over the short study period.

Data availability note. Due to limited availability of annual building-height data for the study area, we used CNBH-10m (2020) as a fixed height basemap and combined it with year-specific boundaries to construct proxy indicators. Future work could incorporate annual height products (e.g., Evolving Cityscapes, 2018–2023) to more directly quantify year-to-year height dynamics and conduct cross-product validation.

### 2.5 Spatial auto-correlation model

Spatial auto-correlation reflects the spatial dependence and heterogeneity of certain indicators due to geographical location or adjacency, and it is used to measure the spatial distribution structure of each indicator in the regional system [51]. Based on the global spatial auto-correlation test (Equation (11)) for spatial clustering, this study uses Local Moran’s I Analysis (LISA) of local spatial auto-correlation (Equation (12)) to identify the spatial clustering pattern of urbanization in Zhangjiakou City [52], and it uses Moran’s I index to reflect the spatial agglomeration law of the urbanization level [53].

Global spatial auto-correlation:


$$I_{Global}=\frac{\sum_{i=1}^{n}\sum_{j=1}^{n}W_{ij}(x_{i}-{\overset{―}{x}}_{i})(x_{j}-{\overset{―}{x}}_{j})}{S^{2}\sum_{i=1}^{n}\sum_{j=1}^{n}W_{ij}}$$ (11)

Here, *n* is the number of counties and districts, *W*_*ij*_ is the spatial weight matrix, *x*_*i*_ and *x*_*j*_ are the urbanization levels of Zhangjiakou City in each dimension, *i* and *j* are the average values of the urbanization levels in each dimension, and *S*^*2*^ is the variance in the urbanization level. If *I*_*Global*_ >0, it means that the indicator is spatially positively correlated with the urbanization level of Zhangjiakou City; if *I*_*Global*_ <0, it means that the indicator is spatially negatively correlated with the urbanization level.

Local spatial auto-correlation:


$$I_{Local}=\frac{\left(x_{i}-{\overset{―}{x}}_{\;}\right)}{S^{2}}\sum_{i\neq j}W_{ij}(x_{i}-{\overset{―}{x}}_{\;})$$ (12)

According to the *I*_*Local*_ results, areas can be divided into four types: high-high, high-low, low-high, and low-low areas. When *I*_*Local*_ >0, it denotes high-high or low-low areas, representing spatial positive correlation. When *I*_*Local*_ <0, it denotes low-high or high-low areas, representing spatial negative correlation. When *I*_*Local*_ = 0, it means that the difference in urbanization level is not significant.

Significance for Global Moran’s I and Local Moran’s I (LISA) was assessed using permutation-based pseudo p-values in GeoDa. We used 999 permutations as the main specification and further conducted robustness checks under alternative permutation settings. Although the exact significance of some marginal local units varied across permutation counts, especially at lower permutation levels, the core high-high clustering pattern remained stable.

### 2.6 Geographic detector model

The geographic detector model is a statistical method for detecting spatial heterogeneity and revealing its driving forces [54], as shown in Equation (13):


$$q=1-\frac{1}{{N\sigma }^{2}}\sum_{i=1}^{L}N_{i}\overset{2}{\underset{i}{\sigma }}$$ (13)

Here, *q* represents the degree to which the driving factor explains the urbanization level; *L* represents the driving factor; *N* and *σ*^*2*^ represent the number of units and variance in the research object, respectively; and *N*_*i*_ and *σ*_*i*_^*2*^ represent the number of spatial units and variance in layer *i*, respectively.

GeoDetector analysis (dimension vs. indicator levels). We applied GeoDetector to quantify the explanatory power (*q*-statistic) of potential determinants of urbanization from two hierarchical levels: (i) the dimension level (population, economic, spatial, and ecological urbanization) and (ii) the indicator level (X_1_–X_17_). For each year, GeoDetector was used to evaluate how these factors explain the spatial heterogeneity of the dependent variable (overall urbanization level). Given the small number of areal units (n = 16), continuous explanatory variables were discretized into four quantile-based strata (quartiles) to ensure balanced group sizes and to avoid extremely small strata that may inflate q under small n. Results are reported as q-statistics and rankings and are interpreted as explanatory diagnostics rather than causal effects.

## 3. Results

### 3.1 Characteristics of urbanization Level changes in Zhangjiakou City

**Fig 3:**
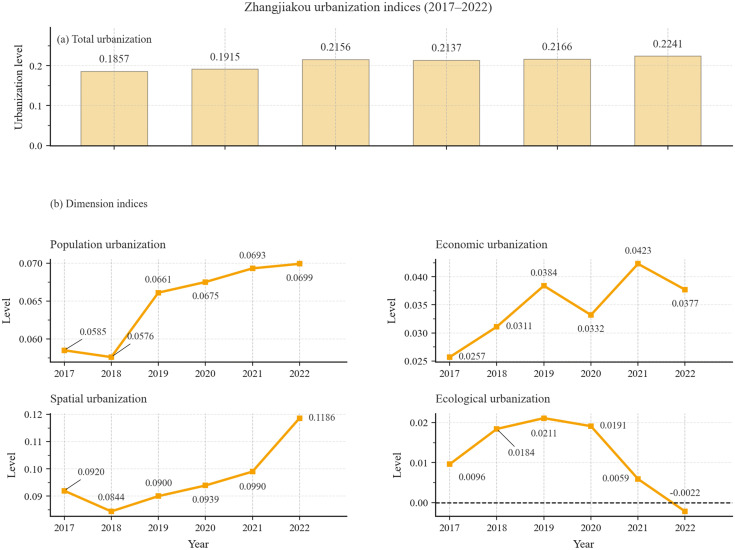
Temporal variation characteristics of urbanization level in Zhangjiakou City during the preparation period of the Winter Olympics. **(a)** This figure depicts the changes in the total urbanization level of Zhangjiakou City. **(b)** These four graphs respectively represent the changes in the urbanization level of Zhangjiakou City in four dimensions: population, economy, spatial and ecology.

As shown in Fig 3, Zhangjiakou’s overall urbanization level fluctuated but trended upward over 2017–2022, rising from 0.1857 in 2017 to 0.2241 in 2022, an increase of 20.68%. At the dimension level, economic urbanization grew most markedly, increasing from 0.0257 to 0.0377 (+46.59%). Over the same period, built-environment and population urbanization also increased despite short-term fluctuations, while ecological urbanization followed an inverted-U trajectory and declined sharply by the end of the study period. Overall, these results indicate that urbanization during the preparation period was multidimensional but uneven, with gains in population, economic, and built-environment urbanization accompanied by ecological deterioration.

**Fig 4:**
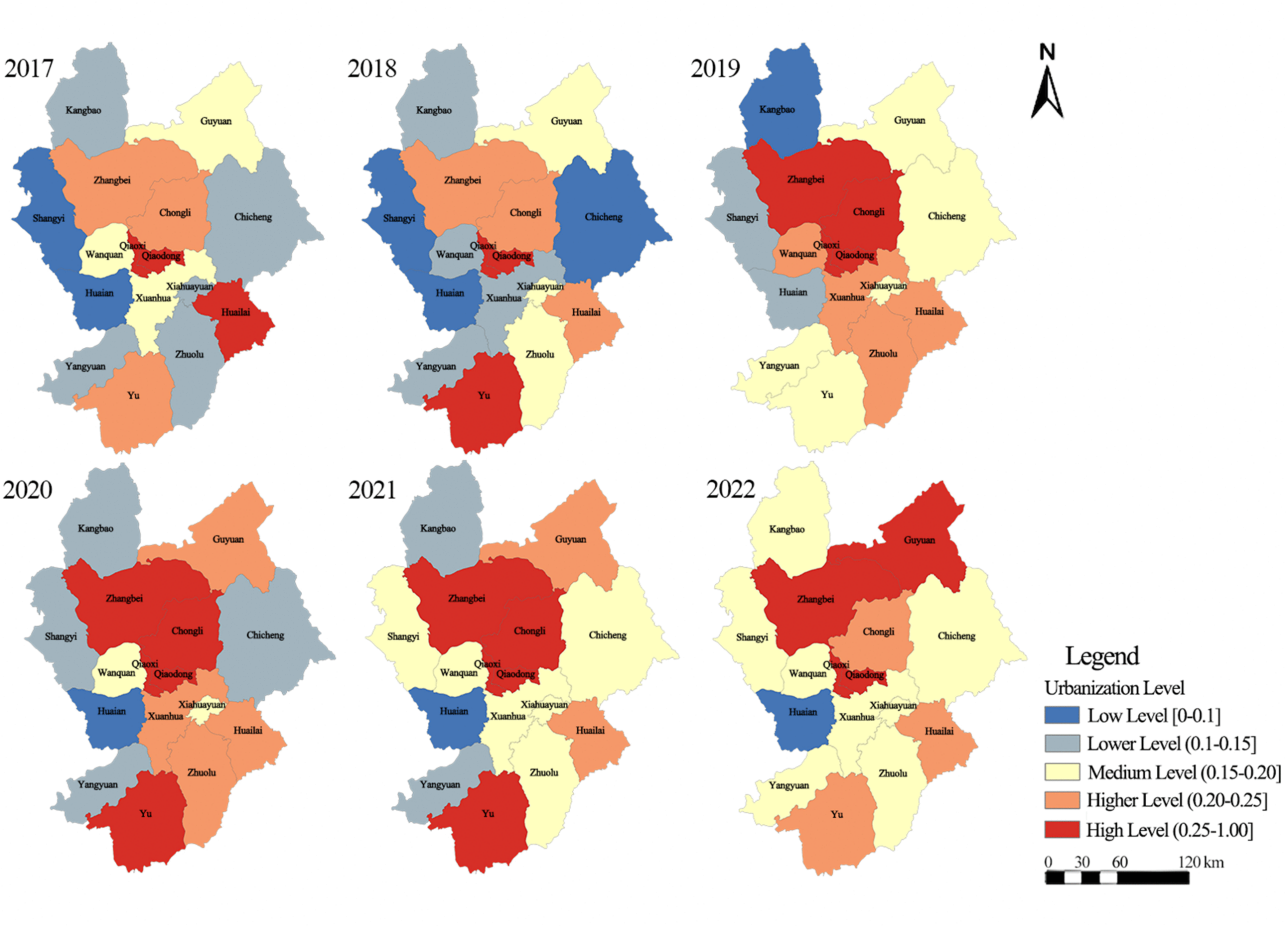
Spatial-temporal evolution trend of urbanization in Zhangjiakou City from 2017 to 2022. The administrative boundary vector data used in this figure are from the Resource and Environment Science and Data Center, Chinese Academy of Sciences.

To characterize the spatial evolution of urbanization, the 16 districts and counties were classified into five tiers using a fixed-break scheme (Fig 4; Table A1 in S1 Appendix). Three broad tendencies can be identified. First, Qiaodong District and Qiaoxi District remained stable urban cores throughout the study period. Second, higher-level urbanization expanded gradually from the central urban area toward the northern region, especially after 2019, with Chongli District, Zhangbei County, and later Guyuan County showing substantial upgrading. Third, the number of districts and counties reaching medium-level urbanization or above increased markedly, although improvement remained uneven across regions. At the regional level, the share of units at medium level or above rose from 100%, 50%, and 40% in the central urban area, northern region, and southern region in 2017 to 100%, 100%, and 80% in 2022, respectively. Overall, these patterns indicate a northward extension of higher-level urbanization rather than a balanced citywide transition.

Qiaodong District and Qiaoxi District maintained persistently high urbanization levels throughout 2017–2022, serving as stable urban cores. Using the 2017 baseline classification, Huailai County was initially categorized as a high-level urbanization area, and 67% of districts/counties were at medium level or above. In 2018, Yu County upgraded to the high-level category, whereas Huailai County shifted down to the higher-level category; meanwhile, Chongli District and Zhangbei County entered the higher-level category, and the share of units at medium level or above was 60%. In 2019, Chongli District and Zhangbei County further upgraded to the high-level category, while Wanquan District and Xuanhua District rose from lower-level to higher-level urbanization; the proportion of units at medium level or above increased to 80%. During 2020–2021, Chongli District, Zhangbei County, and Yu County were consistently classified as high-level areas, and Guyuan County advanced to the higher-level category; the share of units at medium level or above was 67% in 2020 and 80% in 2021. By 2022, Zhangbei County and Guyuan County reached the high-level category, whereas Chongli District shifted to the higher-level category, and the proportion of units at medium level or above increased to 93%. Regionally, the share of units at medium level or above in the central urban area, northern region, and southern region increased from 100%, 50%, and 40% in 2017 to 100%, 100%, and 80% in 2022, respectively. Overall, high-level urbanization agglomerations expanded from the central urban area toward the northern region, accompanied by a marked rise in the number of medium-level units.

At the district/county scale, urbanization trajectories differed markedly across regions. In the central urban region, urbanization levels were generally high but fluctuated substantially over time. Qiaodong District still recorded a net increase of 23.72% between 2017 and 2022, whereas Qiaoxi District showed a net decline of 9.90% over the same period. Chongli District reached a temporary peak during the venue-construction stage in 2020, but its urbanization level in 2022 was only 3.14% higher than that in 2017, indicating limited net gain over the full period.

By contrast, the northern region showed a stronger upgrading trend. Zhangbei County and Guyuan County increased by 46.42% and 40.11%, respectively, between 2017 and 2022, despite short-term fluctuations. Improvement was also observed in the southern region, but to a lesser extent. For example, Yu County recorded a net increase of 19.68% over the study period. Overall, these contrasts suggest that urbanization growth during the preparation period was strongest in the northern region, remained unstable in the central urban area, and was comparatively weaker in the south.

To further examine whether these spatial differences were statistically clustered rather than randomly distributed, spatial autocorrelation analysis was conducted for each dimension.

**Fig 5:**
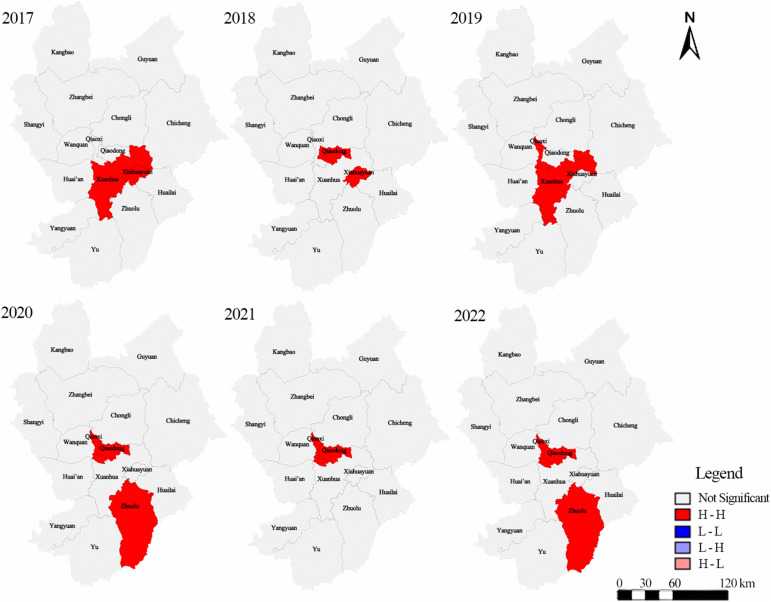
LISA cluster diagram of economic urbanization in Zhangjiakou City from 2017 to 2022. The administrative boundary vector data used in this figure are from the Resource and Environment Science and Data Center, Chinese Academy of Sciences.

**Fig 6:**
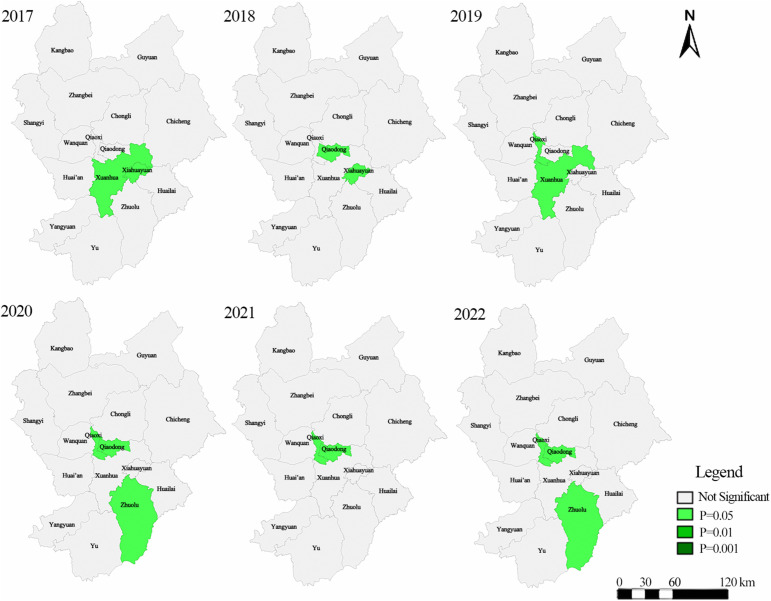
LISA significance diagram of economic urbanization in Zhangjiakou City from 2017 to 2022. The administrative boundary vector data used in this figure are from the Resource and Environment Science and Data Center, Chinese Academy of Sciences.

As shown in Table 3, economic urbanization exhibited significant positive global spatial autocorrelation throughout 2017–2022 (Global Moran’s I > 0, p < 0.05), indicating a clustered spatial pattern. By contrast, the other dimensions did not show statistically significant global spatial autocorrelation in most years. We therefore further examined the local spatial association of economic urbanization using Local Moran’s I (LISA), and the results are summarized in Table 3 and mapped in Fig 5 and Fig 6.

**Table 3:** Urbanization results of Zhangjiakou City from 2017 to 2022.

Dimensions	Year	*I*_*Global*_	*Z*	*P*
Population Urbanization	2017	−0.1084	−0.2790	0.7803
	2018	0.2047	1.8007	0.0718
	2019	0.2702	2.2601	0.0238
	2020	0.2618	2.2125	0.0269
	2021	−0.1424	−0.5085	0.6111
	2022	−0.1465	−0.5404	0.5890
Economic Urbanization	2017	0.3331	2.6819	0.0073
	2018	0.3151	2.5455	0.0109
	2019	0.3175	2.5401	0.0111
	2020	0.3144	2.5260	0.0115
	2021	0.3344	2.6981	0.0070
	2022	0.3144	2.5260	0.0115
Spatial Urbanization	2017	0.2721	2.3039	0.0212
	2018	0.0111	0.5493	0.5828
	2019	0.1370	1.3498	0.1771
	2020	−0.0031	0.4264	0.6698
	2021	−0.0725	−0.0389	0.9690
	2022	0.3532	2.9048	0.0037
Ecological Urbanization	2017	0.1983	1.7646	0.0776
	2018	0.2725	2.3106	0.0209
	2019	0.0650	0.8871	0.3750
	2020	0.2020	1.8452	0.0650
	2021	0.0698	0.9398	0.3473
	2022	0.1909	1.7236	0.0848

Local Moran’s I results (Table 4) show that significant local clustering of economic urbanization was concentrated in the core districts, especially Qiaodong District and Qiaoxi District, which repeatedly appeared as significant clusters in 2018–2022. Early significant areas included Xiahuayuan District and Xuanhua District (2017–2019), whereas Zhuolu County emerged as a significant cluster in the later period (2020 and 2022), suggesting a partial outward extension of local clustering beyond the urban core.

**Table 4:** Results of $I_{Local}$ of Zhangjiakou City’s economic urbanization from 2017 to 2022.

Year	District/County	*I*_*Local*_	*P*
2017	Xiahuayuan District	0.1405	0.0330
	Xuanhua District	0.2161	0.0330
2018	Qiaodong District	1.2847	0.0500
	Xiahuayuan District	0.2177	0.0150
2019	Qiaoxi District	0.7810	0.0270
	Xuanhua District	0.1778	0.0350
2020	Qiaodong District	1.1691	0.0490
	Qiaoxi District	0.7224	0.0460
	Zhuolu County	0.4584	0.0500
2021	Qiaodong District	1.7358	0.0340
	Qiaoxi District	0.7259	0.0230
2022	Qiaodong District	1.1691	0.0490
	Qiaoxi District	0.7224	0.0460
	Zhuolu County	0.4584	0.0500

The LISA results indicate that economic urbanization in Zhangjiakou City was characterized by statistically significant high-high (H-H) clustering over 2017–2022 (pseudo p ≤ 0.05, permutation test). No significant low-low (L-L) clusters or spatial outliers (high-low or low-high) were detected at the 5% level. Specifically, significant H-H clusters were identified in Xuanhua District and Xiahuayuan District in 2017; Qiaodong District and Xiahuayuan District in 2018; Qiaoxi District, Xuanhua District, and Xiahuayuan District in 2019; Qiaodong District, Qiaoxi District, and Zhuolu County in 2020; Qiaodong District and Qiaoxi District in 2021; and Qiaodong District, Qiaoxi District, and Zhuolu County in 2022. Robustness checks using alternative permutation settings show that, although the exact significance of some marginal local units varied across years and permutation counts, the core high-high agglomeration pattern remained stable. In particular, the significant clusters consistently remained within the same core set of districts/counties, namely Qiaodong, Qiaoxi, Xiahuayuan, Xuanhua, and Zhuolu. This pattern suggests that the urbanization benefits associated with the preparation period were geographically concentrated rather than evenly diffused across Zhangjiakou.

### 3.2 Analysis of driving factors of urbanization in Zhangjiakou City

This study employed the GeoDetector method to assess the extent to which factors at the dimension and indicator levels explain the spatial differentiation of urbanization in Zhangjiakou City. The results are reported in Tables 5 and 6. As shown in Table 4, at the dimension level, the ranking of explanatory power varied only modestly over time. Economic urbanization most consistently showed the highest q-values, while ecology generally remained the weakest explanatory dimension. Although the spatial dimension temporarily exceeded the economic dimension in 2018, the overall pattern across the six years indicates that economic urbanization provided the strongest and most stable basis for explaining the spatial heterogeneity of composite urbanization.

**Table 5:** Identification results of driving factors at the dimension level.

Urbanization Dimension	2017		2018		2019		2020		2021		2022	
	*q*	*p*	*q*	*p*	*q*	*p*	*q*	*p*	*q*	*p*	*q*	*p*
Population Urbanization	0.6727	< 0.05	0.4880	< 0.05	0.6326	< 0.05	0.6450	< 0.05	0.4649	< 0.05	0.4680	< 0.05
Economic Urbanization	0.7288	< 0.05	0.7400	< 0.05	0.7603	< 0.05	0.7377	< 0.05	0.4921	< 0.05	0.6423	< 0.05
Spatial Urbanization	0.5000	< 0.05	0.7598	< 0.05	0.5662	< 0.05	0.6943	< 0.05	0.4576	< 0.05	0.1187	< 0.05
Ecological Urbanization	0.3222	< 0.05	0.2214	< 0.05	0.4919	< 0.05	0.2018	< 0.05	0.2085	< 0.05	0.2848	< 0.05

**Table 6:** Identification results of driving factors at the indicator level.

Urbanization Dimension	X	2017		2018		2019		2020		2021		2022	
		*q*	*p*	*q*	*p*	*q*	*p*	*q*	*p*	*q*	*p*	*q*	*p*
Population Urbanization	X_1_	0.8302	< 0.001	0.8425	< 0.001	0.9001	< 0.001	0.8266	< 0.001	0.7393	< 0.001	0.7444	< 0.001
	X_2_	0.8273	< 0.001	0.7939	< 0.001	0.8385	< 0.001	0.7426	< 0.001	0.8038	< 0.001	0.9008	< 0.001
	X_3_	0.8714	< 0.001	0.8632	< 0.001	0.6567	< 0.001	0.8260	< 0.001	0.8562	< 0.001	0.8385	< 0.001
	X_4_	0.7918	< 0.001	0.9073	< 0.001	0.7995	< 0.001	0.8263	< 0.001	0.9571	< 0.001	0.8170	< 0.001
Economic Urbanization	X_5_	0.7935	< 0.001	0.6461	< 0.001	0.6459	< 0.001	0.6039	< 0.001	0.6868	< 0.001	0.5752	< 0.001
	X_6_	0.7096	< 0.001	0.6838	< 0.001	0.7394	< 0.001	0.5421	< 0.001	0.3333	< 0.001	0.7225	< 0.001
	X_7_	0.4108	< 0.001	0.3707	< 0.001	0.3762	< 0.001	0.3737	< 0.001	0.3512	< 0.001	0.2429	< 0.001
	X_8_	0.4610	< 0.001	0.9321	< 0.001	0.5846	< 0.001	0.5132	< 0.001	0.6162	< 0.001	0.4979	< 0.001
	X_9_	0.8349	< 0.001	0.7032	< 0.001	0.8066	< 0.001	0.8585	< 0.001	0.7546	< 0.001	0.8135	< 0.001
Spatial Urbanization	X_10_	0.6828	< 0.001	0.8227	< 0.001	0.6758	< 0.001	0.3760	< 0.001	0.4978	< 0.001	0.3905	< 0.001
	X_11_	0.5798	< 0.001	0.5045	< 0.001	0.5819	< 0.001	0.6651	< 0.001	0.4200	< 0.001	0.5080	< 0.001
	X_12_	0.8363	< 0.001	0.4133	< 0.001	0.7879	< 0.001	0.8453	< 0.001	0.4200	< 0.001	0.8736	< 0.001
	X_13_	0.7357	< 0.001	0.8880	< 0.001	0.7641	< 0.001	0.8540	< 0.001	0.8875	< 0.001	0.8532	< 0.001
Ecological Urbanization	X_14_	0.1541	< 0.001	0.7090	< 0.001	0.8630	< 0.001	0.6727	< 0.001	0.4520	< 0.001	0.5806	< 0.001
	X_15_	0.5460	< 0.001	0.5473	< 0.001	0.4971	< 0.001	0.6968	< 0.001	0.7268	< 0.001	0.6638	< 0.001
	X_16_	0.8160	< 0.001	0.2414	< 0.001	0.2885	< 0.001	0.2892	< 0.001	0.5086	< 0.001	0.6323	< 0.001
	X_17_	0.5175	< 0.001	0.8819	< 0.001	0.9128	< 0.001	0.5632	< 0.001	0.3624	< 0.001	0.4648	< 0.001

In Table 6, there are 17 indicators at the indicator layer were evaluated using GeoDetector, and the results are presented. At the indicator level, the specific ranking of variables varied across years, but several indicators appeared repeatedly among the strongest explanatory factors. In particular, urban economic density (X_13_), per capita urban road area (X_12_), population density distribution (X_2_), and total urban population (X_3_) showed the most stable explanatory power across the study period. Night-time light intensity (X_9_) also performed strongly in some years, especially in the earlier period. Taken together, these results suggest that economic concentration, infrastructure provision, and demographic agglomeration formed the most consistent explanatory basis of urbanization heterogeneity during the Olympic preparation period. Rather than merely reaffirming the general role of transport in urban development, these findings reveal how infrastructure provision, economic concentration, and demographic agglomeration jointly shaped differentiated urbanization outcomes within a medium-sized co-host city.

Overall, across 2017–2022, the GeoDetector results indicate that urban economic density (X_13_), per capita urban road area (X_12_), population density distribution (X_2_), and total urban population (X_3_) were the indicators most strongly associated with the spatial heterogeneity of urbanization in Zhangjiakou. These findings point to the importance of infrastructure and agglomeration conditions during the Olympic preparation period, but they do not on their own identify the independent causal effect of the Winter Olympics.

## 4. Discussion

During the preparation period for the Beijing 2022 Winter Olympics, Zhangjiakou City experienced an overall increase in its urbanization level, with the composite urbanization index in 2022 being 20.68% higher than that in 2017. This increase was driven mainly by growth in the population, economic, and built-environment dimensions, which rose by 19.60%, 46.59%, and 28.97%, respectively, whereas ecological urbanization declined substantially over the same period. These findings indicate that urbanization in Zhangjiakou was not a uniform development dividend, but a multidimensional process in which gains in some dimensions were accompanied by environmental trade-offs. This pattern is broadly consistent with previous studies suggesting that mega-event preparation may coincide with urban construction, economic adjustment, and spatial expansion in host or event-related cities [55–57]. At the same time, the wider literature remains mixed. While some studies report positive demographic or spatial effects, others find limited economic gains or even short-term decline [58]. In Zhangjiakou, the preparation period overlapped with a series of local policy and investment initiatives, including the “Zhangjiakou Ice-Snow Industry Development Plan (2019-2025)” and related infrastructure and industrial upgrading measures across districts and counties [59,60]. These developments likely contributed to economic growth, construction land demand, and outward spatial development to some extent [61]. Meanwhile, the marked decline in the ecological dimension suggests that Olympic-related construction and associated urban expansion also generated environmental pressure. Similar ecological trade-offs have been documented in other mega-event contexts, such as the 2014 Sochi Winter Olympics [62]. In Zhangjiakou, venue construction may have reduced vegetation cover, while the COVID-19 pandemic, ecological restoration projects, and anthropogenic emission-reduction interventions may also have altered environmental conditions and introduced new trade-offs [63–66]. Accordingly, the findings are more appropriately interpreted as multidimensional urbanization dynamics during the Olympic preparation period than as direct causal proof of an independent Olympic effect. It is also important to clarify the role of the CRITIC method in this study. CRITIC was used to derive data-driven weights for heterogeneous indicators in the multidimensional urbanization framework, thereby improving comparability across years and across districts/counties. Its function was not to explain spatial mechanisms directly, but to provide the measurement basis for the composite urbanization index, upon which the subsequent spatial pattern and explanatory analyses were built.

Urbanization change during the preparation period was also spatially uneven across Zhangjiakou. The strongest changes were concentrated in the central urban region, where real-estate activity, infrastructure concentration, and Olympic-related facilities were more intensively clustered, while some southern and less-connected counties showed comparatively weaker improvement. This indicates that the preparation period did not generate a uniform urbanization effect across the city. The central urban region, comprising Qiaodong District, Qiaoxi District, and Chongli District, served as the main hosting area and also constituted a high-high economic agglomeration zone within Zhangjiakou. However, its development trajectory remained constrained by pre-existing structural conditions. Prior to the Winter Olympics, heavy industry dominated the local economy, and the region’s economic structure has been described as relatively underdeveloped [67]. Notably, the Winter Olympics did not produce a sustained boost to the real-estate market across the central urban region. For example, the urbanization level of Qiaoxi District decreased by 9.90% between 2017 and 2022, contrasting with findings from Pyeongchang, where housing-market effects were found to be more persistent [68]. Several factors may account for this difference, including the outdated industrial structure of the central urban region [69], labor outflows associated with uneven development within the Beijing-Tianjin-Hebei region [70], macroeconomic policies aimed at curbing real-estate bubbles [71,72], and intensified downward pressure under the global pandemic [73,74]. At the same time, infrastructure improvements may still have generated short-term local gains. The opening of the Beijing–Zhangjiakou high-speed railway and Olympic-related construction likely improved accessibility and stimulated localized urbanization growth [75]. For instance, Chongli District reached its highest urbanization level in 2020 during the venue construction stage, representing a 14% increase relative to 2017, while Xiahuayuan District recorded a 34% increase in 2019 compared with 2017 after the opening of the Beijing–Zhangjiakou high-speed railway. However, these gains were not necessarily sustained over the full study period. The urbanization level of Chongli District in 2022 was only 3.14% higher than that in 2017, indicating limited net gain after the peak construction phase. In addition, pandemic-related restrictions severely damaged tourism and service activities [76–78], suggesting that the large investments associated with hosting the Winter Olympics may have exceeded the realized short-term benefits during this period [30]. Taken together, these patterns suggest that Olympic-related opportunities in the central urban region were filtered through local industrial structure, regional inequality, macroeconomic regulation, and pandemic-related shocks, rather than producing a uniform direct effect across all districts.

Urbanization growth in the northern region appears to have been more closely associated with the alignment between Olympic-related investment and existing local development conditions. Counties such as Zhangbei and Guyuan combined infrastructure upgrading with tourism development and the expansion of emerging industries, including big data and renewable energy [36,79,80]. Compared with the central urban region, the northern region appears to have been better positioned to translate Olympic-related policy support into broader economic and spatial growth, partly because investment could be linked more directly to local resource endowments and sectoral opportunities. As a result, the overall urbanization level in the northern region increased substantially. For example, the urbanization level of Zhangbei County in 2022 was 46.42% higher than that in 2017, and the share of counties and districts in the northern region reaching medium-level urbanization or above increased from 50% in 2017 to 100% in 2022. These findings are broadly consistent with previous research showing that ice-snow tourism and related industries may contribute to regional development and poverty reduction under suitable local conditions [61,81]. At the same time, the northern case should not be interpreted as evidence of a uniform Olympic dividend. Rather, it suggests that Olympic-related investment was most effective where it aligned with pre-existing locational advantages, natural-resource endowments, and sectoral development opportunities. This further reinforces the spatially selective character of urbanization in Zhangjiakou during the preparation period.

The contribution of this study is therefore not merely to reconfirm the well-established role of transport and infrastructure in urban development. Rather, by examining Zhangjiakou as a medium-sized Olympic co-host city on the Beijing metropolitan periphery, the study shows that urbanization during the preparation period was multidimensional, spatially uneven, and ecologically differentiated. Gains in population, economic, and built-environment urbanization did not translate into balanced citywide improvement, and ecological decline remained a significant trade-off. These findings extend the mega-event urbanization literature in three respects. First, they shift attention from core metropolitan hosts to a medium-sized co-host city, where event-related opportunities are mediated more strongly by regional inequality, local industrial structure, and limited agglomeration capacity. Second, they show that urbanization should not be evaluated through a single outcome such as GDP, land expansion, or tourism growth alone, but through multiple dimensions that may evolve asynchronously. Third, they demonstrate that the gains and costs of mega-event preparation were distributed selectively across districts and counties, rather than diffusing evenly throughout the city. In this sense, Zhangjiakou illustrates how mega-event preparation in a medium-sized co-host city may function less as a uniform driver of urbanization than as a contextual catalyst whose effects depend heavily on local development geography.

This study has several limitations. First, although the four-dimensional framework of population, economy, built-environment, and ecology captures major structural aspects of urbanization, it does not directly reflect governance capacity, public service provision, social inequality, housing affordability, or subjective well-being. The findings should therefore be interpreted as a multidimensional assessment of structural urbanization change rather than a complete evaluation of urban development quality. Second, annual building-height surfaces were unavailable, so the 2020 CNBH-10 m layer was used as a fixed height baseline, which may underestimate interannual vertical change. Third, GeoDetector results may be sensitive to discretization under a small sample size; accordingly, the reported results are interpreted as explanatory associations rather than definitive causal mechanisms. Fourth, the present design does not isolate the independent net effect of hosting the Winter Olympics from other concurrent processes, such as infrastructure upgrading, industrial restructuring, regional spillovers, and the COVID-19 pandemic. Finally, as the study focuses on Zhangjiakou as a medium-sized Olympic co-host city in a specific regional context, the findings should be generalized with caution.

## 5. Conclusions

This study integrated multi-source remote sensing and socio-economic data and applied the CRITIC method to construct a multidimensional urbanization evaluation system for Zhangjiakou City. Spatial autocorrelation analysis and GeoDetector were then used to examine the spatiotemporal evolution of urbanization and the factors associated with its spatial heterogeneity across districts and counties during the preparation period for the Beijing Winter Olympics. The main findings are as follows:

(1) From 2017 to 2022, the overall urbanization level of Zhangjiakou City increased. Population, economic, and built-environment urbanization rose over the study period, whereas ecological urbanization declined.
(2) The spatial pattern of urbanization shifted over time: high-level urbanization areas expanded northward from the central urban core, and the number of medium-level urbanization units increased markedly.
(3) Economic urbanization exhibited significant spatial clustering throughout 2017–2022 (Global Moran’s I; see Table 2), with local high-high (H-H) clusters concentrated in the central urban area and, from 2019 onward, also emerging in Zhuolu County in the south (LISA; see Table 3 and Fig 5).
(4) At the dimension level, the economic component consistently showed the strongest explanatory power for overall urbanization during the study period. At the indicator level, urban economic density, per capita urban road area, population density distribution, and total urban population were the most stable explanatory correlates (Tables 4 and 5).

These findings indicate that urbanization in Zhangjiakou during the Olympic preparation period was multidimensional, spatially uneven, and ecologically differentiated, rather than a uniform development dividend. Methodologically, the study demonstrates that the CRITIC method can provide a useful weighting basis for multidimensional urbanization evaluation when heterogeneous remote-sensing and socio-economic indicators are combined, while spatial autocorrelation analysis and GeoDetector can then be used to interpret the resulting spatial patterns and explanatory associations. The main contribution of this study is not simply to reconfirm the well-established role of infrastructure and accessibility in urban development, but to show that in a medium-sized Olympic co-host city, the gains and costs of mega-event preparation were distributed selectively across dimensions and across space.

Based on these results, several policy implications can be drawn for post-Winter Olympics development. Development strategies should be differentiated according to regional conditions. The northern region could further strengthen the ice-and-snow tourism system while advancing new energy and related emerging industries on existing foundations. The central urban area should accelerate industrial upgrading, implement more competitive talent-attraction and retention policies, enhance energy collaboration with the northern region, and leverage its industrial base to support high-tech development. The southern region should prioritize improvements in rural infrastructure, optimize folk-tourism offerings, and promote deeper integration with ice-and-snow sports. Continued efforts to consolidate poverty alleviation outcomes and to balance development gains with ecological protection are also warranted.

## Supporting information

S1 Data Minimal supporting dataset. (ZIP)

S1 Appendix Appendix Table A1. (DOCX)
