# Supplementary material for: Urbanization change in a mega-event preparation context: A multidimensional assessment of Zhangjiakou, a medium-sized co-host city of the Beijing 2022 Winter Olympics
Source: PLoS One. 2026 May 6;21(5):e0339708. doi: 10.1371/journal.pone.0339708 (PMC13148699; doi:10.1371/journal.pone.0339708)
Supplement: S1 Data — (ZIP) [file pone.0339708.s001.zip › Minimal Supporting Dataset/S4_Metadata.pdf]

The supporting files provide the minimal dataset required to reproduce the main findings of this study. The data cover 16 districts/counties in Zhangjiakou City for the period 2017–2022 and include the indicator dataset, composite urbanization evaluation results, spatial autocorrelation results, and GeoDetector results.

S1_Data.xlsx

This file contains the district- and county-level annual dataset used for the multidimensional urbanization evaluation in this study (2017–2022). It includes the original indicator values, the normalized values, the CRITIC weighting process, the dimension-level scores, and the final composite urbanization index. These data directly support the urbanization evaluation results reported in the manuscript, including Figures 3 and 4 and the related tables.

S2_Moran's I.zip

This compressed file contains the GeoDa output files and map images for the district- and county-level urbanization data. It includes the results of Global Moran's I for the four urbanization dimensions and the Local Moran's I (LISA) results for economic urbanization. These files support the spatial autocorrelation analysis reported in the manuscript, including Table 3, Table 4, Figure 5, and Figure 6.

S3_GeoDetector.zip

This file contains the GeoDetector results used to assess the extent to which different urbanization dimensions and individual indicators are associated with the spatial heterogeneity of overall urbanization across districts and counties. It includes the q-statistics and related significance results at both the dimension level and the indicator level. These results support the explanatory analysis reported in the manuscript, including Tables 5 and 6.

S4_Metadata.pdf
Content of Supporting Data.
