# Supplementary material for: Urbanization change in a mega-event preparation context: A multidimensional assessment of Zhangjiakou, a medium-sized co-host city of the Beijing 2022 Winter Olympics
Source: PLoS One. 2026 May 6;21(5):e0339708. doi: 10.1371/journal.pone.0339708 (PMC13148699; doi:10.1371/journal.pone.0339708)
Supplement: S1 Appendix — (DOCX) [file pone.0339708.s002.docx]

**Appendix**

In the appendix we list the “Changes in Urbanization Level of Various Districts and Counties in Zhangjiakou City during the Winter Olympics” as Table A1.

Table A1: Changes in Urbanization Level of Various Districts and Counties in Zhangjiakou City during the Winter Olympics.

| Regions | District/County | 2017 | 2018 | 2019 | 2020 | 2021 | 2022 |
| --- | --- | --- | --- | --- | --- | --- | --- |
| Northern Region | Chicheng County | 0.1461 | 0.0736 | 0.1655 | 0.1418 | 0.1598 | 0.1734 |
|  | Guyuan County | 0.1803 | 0.1847 | 0.1617 | 0.2178 | 0.2288 | 0.2526 |
|  | Kangbao County | 0.1057 | 0.1224 | 0.1176 | 0.1045 | 0.1315 | 0.1923 |
|  | Shangyi County | 0.0858 | 0.0893 | 0.1393 | 0.1269 | 0.1522 | 0.1845 |
|  | Zhangbei County | 0.2026 | 0.2260 | 0.2565 | 0.2545 | 0.2583 | 0.2967 |
| Central Urban Region | Chongli District | 0.2382 | 0.2236 | 0.2557 | 0.2778 | 0.2574 | 0.2457 |
|  | Qiaodong District | 0.3148 | 0.4344 | 0.3315 | 0.3691 | 0.4223 | 0.3895 |
|  | Qiaoxi District | 0.4293 | 0.3753 | 0.4433 | 0.4526 | 0.3909 | 0.3868 |
|  | Wanquan District | 0.1500 | 0.1263 | 0.2100 | 0.1841 | 0.1751 | 0.1675 |
|  | Xiahuayuan District | 0.1214 | 0.1595 | 0.1838 | 0.1553 | 0.1811 | 0.1815 |
|  | Xuanhua District | 0.1742 | 0.1477 | 0.2103 | 0.2090 | 0.1697 | 0.1710 |
| Southern Region | Huai’ an County | 0.0860 | 0.0938 | 0.1434 | 0.0739 | 0.1060 | 0.0931 |
|  | Huailai County | 0.2504 | 0.2201 | 0.2478 | 0.2397 | 0.2147 | 0.2346 |
|  | Yu County | 0.2071 | 0.2630 | 0.1986 | 0.2741 | 0.2969 | 0.2478 |
|  | Yangyuan County | 0.1322 | 0.1453 | 0.1506 | 0.1367 | 0.1452 | 0.1708 |
|  | Zhuolu County | 0.1478 | 0.1790 | 0.2339 | 0.2009 | 0.1763 | 0.1974 |
